# The Efficacy of an Intensive Lifestyle Modification Program on Psychosocial Outcomes among Rural Women with Prior Gestational Diabetes Mellitus: Six Months Follow-Up of a Randomized Controlled Trial

**DOI:** 10.3390/ijerph18041519

**Published:** 2021-02-05

**Authors:** Jia Guo, Qing Long, Jundi Yang, Qian Lin, James Wiley, Jyu-Lin Chen

**Affiliations:** 1 Xiangya School of Nursing, Central South University, Changsha 410013, China; longqing226@163.com (Q.L.); jundiyang_miumiu@163.com (J.Y.); 2 Department of Nutrition Science and Food Hygiene, Xiangya School of Public Health, Central South University, Changsha 410078, China; linqian@csu.edu.cn; 3 Philip R. Lee Institute for Health Policy Studies, University of California, San Francisco, CA 94143, USA; jwiley124@gmail.com; 4 School of Nursing, University of California, San Francisco, CA 94143, USA; jyu-lin.Chen@ucsf.edu

**Keywords:** gestational diabetes mellitus, lifestyle intervention, stress, social support, quality of life

## Abstract

Women with prior gestational diabetes mellitus (GDM) are at a higher risk of type 2 diabetes and other health issues after delivery. They may have a lower quality of life (QoL), experience more medical-related stress, and need more support than those without it. This study aimed to examine the six-month efficacy of an intensive lifestyle modification program on perceived stress, social support, and QoL among women with prior GDM in rural China. A total of 320 women with prior GDM were randomly assigned to an intervention group (*n* = 160) and a control group (*n* = 160). Participants in the intervention group received an intensive lifestyle modification (ILSM) program, including a series of six biweekly face-to-face sessions and five biweekly phone sessions delivered by trained local health workers. The control group received the usual care. Data about perceived stress, social support, QoL, and HbA1c were collected at baseline, at three months, and at six-month follow-ups. Generalized estimating equation analysis was used to assess the efficacy of the intervention. There were significant improvements in the psychological domain (β = 0.479 ± 0.153, *p* = 0.002) and environmental domain (β = 0.462 ± 0.145, *p* = 0.001) of QoL over six months; there were significant group effects (β = −0.718 ± 0.280, *p* = 0.010) and time effects (β = 0.453 ± 0.211, *p* = 0.032) in physiological domain, and there were significant group effects in the social relations domain (β = −0.669 ± 0.321, *p* = 0.037). The ILSM group had a more pronounced downward trend in HbA1c than the control group (β = −0.050 ± 0.026, *p* = 0.059). The ILSM program can help women with GDM improve their psychological and environmental domain of QoL. It can be recommended as a form of health promotion for improving QoL among women with prior GDM in rural primary care settings in developing countries.

## 1. Introduction

Gestational diabetes mellitus (GDM) is defined as any degree of glucose intolerance that is first observed during pregnancy [1]. The prevalence of GDM has increased by more than 30% within the last two decades, making it an emerging worldwide epidemic [2]. A recent meta-analysis suggests that the total incidence rate of GDM in mainland China is 14.8%, according to the International Association of Diabetes and Pregnancy Study Groups criteria [3]. GDM is associated with an increased risk for fetal and maternal complications, such as preeclampsia and macrosomia, during pregnancy [4].

In the long term, women with GDM have a higher risk of chronic illness after delivery, such as type 2 diabetes (a seven-fold increased risk), hypertension (1.56 times the risk), and obesity (1.30 times the risk) later in life, compared to women without GDM [5,6]. The offspring of women with GDM are also at an increased risk of developing obesity, metabolic syndrome, or type 2 diabetes mellitus (T2DM) [5,7]. Given that women with GDM are at a higher risk for other health issues, an intervention aimed to promote healthy lifestyles and reduce this risk is essential for the health of women and their children.

The postpartum period adds another layer of stress for women with GDM. Besides common postpartum problems, such as sleep deprivation, hormonal changes, and the demands of caring for a newborn [8], women with prior GDM not only need to receive additional screen tests for diabetes, but may worry about the increased risk of diabetes and cardiovascular disease for themselves and their children [9]. When they are not aware of T2DM risk and prevention after delivery, they may experience more serious health problems without attention to prevention [10]. Their psychological stress and quality of life (QoL) may be affected long after pregnancy and delivery [11].

QoL is defined as “how well a person functions in their life and his or her perceived well-being in physiological, psychological, social functioning, and environmental domains of health” [12]. In Western countries, women with GDM report more adverse postpartum QoL and perceive themselves as less healthy than women with normal pregnancies [13]. In Italy and Canada, the QoL of women with GDM remained significantly lower than women with normal glycemic pregnancies even three to five years after delivery [11,14]. Unfortunately, there has been very little research on the postpartum QoL of women with GDM and the impact of interventions on QoL in developing countries.

Perceived stress is the feeling or thought that an individual has about how much stress they experience in a given time period [15]. Although there is little empirical data on the level of perceived stress among women with prior GDM, research reported by Feig et al. reported that the stressors of women with prior GDM generally focused on concerns about their own health and their children’s health.

Social support refers to functions performed for an individual by their social connections with the expectation of a beneficial outcome, such as emotional sustenance, information provision, or instrumental assistance [16]. Women with prior GDM have consistently expressed a strong need for social support from family, health professionals, and peers to make and sustain changes in dietary and physical activity habits [17]. In addition, they tend to feel disconnected from their health care providers after delivery and lack the active involvement of their partners in their health management [18,19].

While physiological outcomes in response to social cognitive theory-based lifestyle interventions are well researched in women with prior GDM [20], the effectiveness of these interventions on perceived stress, social support, and QoL is less known [20,21]. Based on the theory, the psychosocial effects of these lifestyle interventions should be beneficial [22]. One study conducted in the general population of the United States showed that after lifestyle interventions, physiological QoL rebounded quickly at 1–2 months postpartum [23]. However, more studies are needed to measure the magnitude and duration of these effects and impacts on psychosocial outcomes among women with prior GDM.

The aim of this study was to examine the six-month efficacy of social cognitive theory-based lifestyle interventions on psychosocial outcomes (perceived stress, social support, and QoL) and diabetes prevention outcomes (HbA1c) among women with prior GDM in China.

## 2. Materials and Methods

### 2.1. Design, Setting, and Sample

The multisite randomized clinical trial (RCT) has been reported elsewhere and registered in the Chinese Clinical Trial Registry (ChiCTR) (No. ChiCTR1800015023) [24]. To represent different socioeconomic status, lifestyle, and ethnic groups in the Hunan Province, two counties were selected as study sites: one county with a less developed economy and a large ethnic minority population and another county with middle to high economic development and a predominately Han population. The study was conducted in these counties from November 2017 to June 2019. Two hospitals with the largest number of births in the two counties were selected as venues to recruit participants. Potential participants were found using the delivery records from January 2013 to March 2018. Those who met the inclusion and exclusion criteria received a phone call from trained local health workers to ensure the enrolment criteria, explain the purpose of the study, and assess their interests in participation.

The inclusion criteria included (1) women with a history of GDM; (2) women who were 18 years or older; (3) women who were at least six weeks post-partum at the beginning of the study; (4) women who had never been diagnosed with type 2 diabetes or type 1 diabetes; and (5) women who had telephone-access either to family members, friends, or neighbors. The exclusion criteria included (1) women who were currently pregnant; (2) women who had a diabetes diagnosis before pregnancy or after delivery; (3) women who were having difficulties in communication, such as reading or answering the questionnaire, and were unable to understand the aim of the study; and (4) women who had severe psychiatric disorders. The sample size of *n* = 320 (160 in the intervention group and 160 in the control group) was based on the change in insulin resistance in a previous trial [24]. For testing hypotheses about the impact of the intervention on QoL measures, we calculated power based on the G*Power 3 option “ANOVA repeated measures for between group (2 groups) by within group (3 times) interactions” based on F-tests [24]. The null hypothesis is that this interaction is nil and the alternate hypothesis is that the intervention group will show more positive change than the control group. The power of this test for a small effect size (a ratio of variances) of 0.10, a modest correlation of 0.5 between the repeated measures, and an alpha = 0.05 were calculated by G*Power 3 as 0.794. For a higher effect size of 0.20, the power was estimated as 0.99.

This study was approved by the ethical review board of Xiangya School of Nursing, Central South University (IRB #2016034). Anonymity and confidentiality of participants were ensured by assigning a study ID number to all participants. Written informed consent was obtained from all the study participants.

### 2.2. Randomization and Blinding

Randomization was completed at the town level by an independent researcher who was not aware of the numeric code for the towns, using a computerized procedure [25]. After randomly allocating the town into the intervention or control group, the eligible participants from each town were assigned to each group, respectively. The trained local health workers who conducted the intensive lifestyle modification (ILSM) and the participants in this study were not blinded to group assignment because it was not possible. The research assistants who collected the data for evaluating the effectiveness of the intervention were blinded to group assignment.

### 2.3. The Usual Care

The usual care provided in this program utilized current clinical guidelines and recommendations for diabetes prevention [26]. Participants in both groups received the same brochure of diabetes prevention education information. Apart from these contents, no other lifestyle promotion services were provided in the control group.

### 2.4. Intensive Lifestyle Modification (ILSM) Based on Social Cognitive Theory

The detailed ILSM has been reported elsewhere [24]. The intervention group was offered the ILSM, including a series of six biweekly, face-to-face sessions and five biweekly phone sessions delivered by trained local health workers during the three-month intervention. The in-person sessions consisted of six topics: (1) orientation and goal setting, (2) healthy eating patterns, (3) physical activities, (4) stress management, (5) family support on the ILSM and family lifestyle patterns, and (6) relapse prevention and farewell. The telephone maintenance sessions of the ILSM program consisted of four aspects: (1) review of progress toward dietary, physical activity, and goals, (2) identification of challenges faced in achieving goals, (3) assistance with setting new action plans and achievable goals, and (4) encouragement in the achievement of goals. Each group session was 90 min long with 16–20 participants, and each phone session lasted about 20 min.

### 2.5. Outcomes and Measures

All the outcomes for both groups were measured at the research sites at the baseline, three months, and six months. The baseline and follow-up assessments were conducted by trained research assistants who were blinded to the group assignment.

QoL was assessed using the WHOQOL-BREF questionnaire, which is a shortened version of the WHOQOL-100 [27]. It includes 26 questions and covers the physiological, psychological, social relations, and environmental dimensions. The physiological domain of QoL includes questions related to pain and discomfort, dependence on medicinal substances and medical aids, energy and fatigue, mobility, sleep and rest, activities of daily living, and work capacity. The psychological domain of QoL includes questions related to body image and appearance, negative feelings, positive feelings, self-esteem, spirituality or religion or personal beliefs, and thinking, learning, memory, and concentration. The social relations domain of QoL includes questions related to personal relationships, sexual activity, and social support. The environmental domain of QoL includes questions related to financial resources, freedom, physical safety and security, health and social care, accessibility and quality, home environment, opportunities for acquiring new information and skills, participation in and opportunities for recreation or leisure activities, and transportation. The participants scored the items on a scale from 1 to 5, and the raw domain scores were converted to a scale of 4–24 for each domain. A higher score on each domain relates to a better QoL. The Cronbach’s alpha ranged from 0.69 to 0.85 in our sample.

Perceived stress was measured by the perceived stress scale. This scale was developed by Cohen, Kamarck, and Mermelstein [16], and was validated in the Chinese population [28]. The scale is designed to measure the degree to which situations in one’s life are appraised as stressful. There are 14 items with a total score ranging from 0 to 56. Higher scores indicate more stress. A criterion score of 26 indicates a negative impact of stress on physiological and mental health. The Cronbach’s α in our sample was 0.83.

Social support was measured using the social support rating scale (SSRS), which was originally developed in Chinese by Xiao [29]. It consists of 10 items measuring three dimensions: objective support (3 items), subjective support (4 items), and support utilization (3 items). Objective support reflects the degree of actual support received in the past. Subjective support reflects the perceived interpersonal network that an individual can count on. Support utilization refers to the pattern of behavior that an individual utilizes when seeking social support. The score of the objective support domain ranges from 1 to 22, the subjective support domain ranges from 8 to 32, and the support utilization domain ranges from 3 to 12. A higher number means that perceived social support is at a high level. This scale has been widely used in different Chinese communities. The test–retest reliability was 0.92, with the consistency of each item falling between 0.89 and 0.94 [30]. The Cronbach alpha coefficient for the total score was 0.72 in our sample.

HbA1c was assessed in the total blood collected with ethylene diamine tetraacetic acid (EDTA) and was measured by fluorescent affinity immunochromatography method (Wandfo FS-201, Guangzhou, China). HbA1c level reflects the mean glucose concentration over the previous period (approximately 8–12 weeks, depending on the individual), with a normal range of 4%–6% [31]. HbA1c value has been used as the diagnosis criteria of diabetes or prediabetes in clinical guidelines in many countries [1,31].

### 2.6. Fidelity

Fidelity was evaluated using two trained research assistants observing and documenting the delivery of all program components across all sessions. The trained field officers had an instruction manual in which every session was clearly described. The research assistant prompted the session deliverer if any component was not completed.

### 2.7. Data Analysis

Data analysis was performed using the Statistical Product and Service Solutions (version 22.0; SPSS Inc., New York, NY, USA). Data were analyzed according to the intention-to-treat principle with longitudinal data analysis techniques (generalized estimating equation (GEE) analysis). GEE provides robust estimates of main and interaction effects when residuals from repeated measures ANOVA were significantly non-normal as judged by the Kolmogorov–Smirnov test. The last observation carried forward (LOCF) method was used to replace missing data. We summarized baseline sample characteristics using descriptive statistics and compared groups using *t* or χ^2^ tests. A series of GEEs were used to examine the main effects of group (ILSM group vs. control group) and time (baseline, three and six-month follow-ups) and the group × time interaction for each of the outcome variables. Two-side *p* values were reported with a statistical significance level of <0.05.

## 3. Results

### 3.1. Baseline Characteristics and Follow-Up

**Figure 1:**
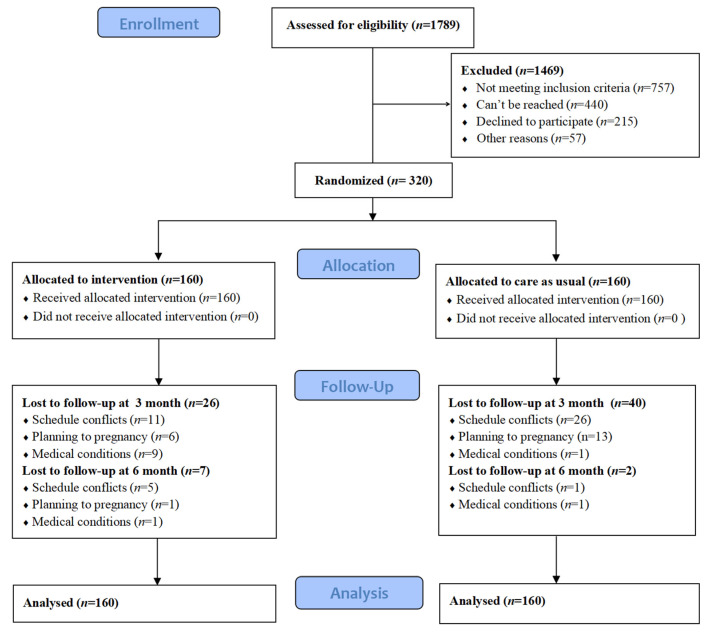
Study design and participant flow.

A total of 1789 women were invited to participate in the study: 757 (42.3%) were excluded due to not meeting the inclusion criteria, 440 (24.6%) could not be reached, 215 (12.0%) declined to participate due to a lack of interest, and 57 (3.2%) had family emergencies or schedule conflicts. The remaining 320 (32.0%) women were recruited, and they provided written informed consent. At the end of the study, 245 women (76.6%) completed all three time measurements over six months (127 in the ILSM group and 118 in the control group) (Figure 1). There were no significant differences between the 245 participants and the 75 participants who missed measurements on demographic and clinical characteristics (*p* > 0.05).

Regarding sociodemographic characteristics, the mean age of the participants was 31.92 years (SD 4.91). About 55.0% were Han nationality, 75.1% of the participants had a senior high school or higher level of education, and 8.3% had a family monthly income greater than $423 per person, which is a low–moderate family income in China. Regarding GDM-related characteristics, the average time after the last delivery upon recruitment for this study was 17.55 months (SD 17.17), and 55.5% were more than 12 months postpartum (225 out of 317). Approximately two-thirds (149 out of 315) of the participants had two or more children. The mean HbA1c was 5.32% (SD 0.43) (see Table 1).

There were no statistically significant differences at baseline between participants of the ILSM group and control group with respect to sociodemographic characteristics (age, ethnicity, education level, occupation, and family monthly income per person), GDM-related characteristics (months after last delivery, parity, or HbA1c), or most of the psychosocial indicators (perceived stress, the total score of social support, subjective support, objective support, support utilization, and social relations domain of QoL) (*p* > 0.05). However, there were significant differences in the physiological, psychological, and environmental domains of QoL (*p* < 0.05) (see Table 1).

### 3.2. The Comparisons of Perceived Stress, Social Support, Four Qol Domains Between the ILSM, and Control Groups from Baseline to the Six-Month Follow Up

There was no significant group by time interaction effects for the ILSM program on perceived stress (β = −0.186 ± 0.560, *p* = 0.739). There was a near significant group by the time interaction effect for subjective support (β = −0.632 ± 0.354, *p* = 0.074). However, there were no significant group by time interaction effects for the ILSM program on objective support (β = 0.135 ± 0.282, *p* = 0.631) and support utilization (β = −0.134 ± 0.142, *p* = 0.345) over six months (see Table 2).

The ILSM group showed significant improvements in two domains of QoL over six months (psychological domain (β = 0.479 ± 0.153, *p* = 0.002) and environmental domain (β = 0.462 ± 0.145, *p* = 0.001)) than the control group. There were significant differences between groups (β = −0.718 ± 0.280, *p* = 0.010) and over time (β = 0.453 ± 0.211, *p* = 0.032) in the physiological domain of QoL. Although the group by the time interaction effect was not significant (β = 0.231 ± 0.143, *p* = 0.105). There was greater improvement in the social relations domain of QoL for the ILSM group in comparison with the control group (β = −0.669 ± 0.321, *p* = 0.037), but the group by time interaction effect was not significant (β = 0.124 ± 0.160, *p* = 0.439) (see Table 2).

### 3.3. The Comparisons of HbA1c between the ILSM and Control Groups from Baseline to the Six-Month Follow up

There was a near significant group by time interaction effect for the ILSM program versus the control group over six months (β = −0.050 ± 0.026, *p* = 0.059) for HbA1c, but both groups showed a clear increase over time (β = 0.211 ± 0.043, *p* = 0.000) (see Table 2).

## 4. Discussion

This study contributed new evidence on the psychosocial effects of social cognitive theory-guided lifestyle interventions aimed to prevent T2DM in rural Chinese women with prior GDM in a randomized study design. In this study, we found these interventions to be effective for improving women’s psychological and environmental domains of QoL over six months. The ILSM program has a potential effect on decreasing the increases in HbA1c, improving the physiological domain of QoL, and improving subjective social support over six months compared to usual care. This study provides a model for implementing a health promotion program among women with prior GDM in rural China.

The participants in the ILSM program showed significant improvement in the psychological domains of QoL over six months compared to usual care. This is likely because the ILSM program employed sessions (such as stress management) targeting improvement of the psychosocial status. In addition, previous research had shown that when the behavioral goals of diabetes management or weight loss were achieved among people with T2DM or obesity, their psychological well-being improved [32]. The ILSM encouraged participants to set and achieve specific behavioral change goals for preventing diabetes in each session [24]. In the group format context, the participants may have felt close collaboration with the group members, moving them towards meaningful goals and contributing to improved psychological domains.

We also found an improvement in the environmental domain of QoL between groups over six months. The environmental domain of QoL includes items related to the home environment, physical safety, the individual’s possibility to access leisure activities, health services, and public transport [27], which are important in the context of the present study. According to Gilbert et al. [33], the environmental domain of QoL among the general Chinese population was improved by providing adequate resources (e.g., health care visits and common rooms with recreation facilities) and a safe environment. In rural China, unlike the public resources provided in big cities, inadequate in-service training, poor health resource distribution, and transportation difficulties are common [34]. The ILSM program provided free systematic health-related lifestyle education, telephone booster sessions, aerobic exercise DVDs, and related transportation reimbursement. Thus, the enhancement of an individual’s possibility to access the health services provided in the ILSM program could increase the environmental domain of QoL in this population.

The trend in improvement in the physiological domain of QoL can be explained by the aspect of the ILSM program that employed sessions on healthy eating patterns and physical activities. This result was in line with the finding that an active lifestyle preserves physical function in women with prior GDM [20,35]. It is noteworthy that both the ILSM group and the control group showed a greater increase in the physiological domain over six months. One possible explanation could be that frequent contact from the health care providers and three free physical examinations may remind and motivate the participants to pay more attention to physical health.

The trend in improvement in the social relation domain of QoL may be due to the sessions (such as family support and family lifestyle patterns) that are incorporated in the ILSM program. These results were consistent with a trend we found in the improvement of subjective support between groups over time. Objective support and support utilization are not easily improved in a short-term period. A significant change might be detected if we followed up for a longer period.

Both the ILSM group and the control group showed an increasing trend of HbA1c over six months, which means this population did have a growing risk of developing diabetes even during a short time period. This finding is in line with the results among women with prior GDM [36]. However, we found an exciting lower rate of increase of HbA1c between the ILSM group compared with the control group over time, which means the ILSM program has the latent capacity to prevent the growing risk of developing diabetes over time among women with prior GDM. Ongoing research will determine if these changes exist in the long term.

The current trial had some weaknesses, which limit the interpretation of these findings. First, only the short-term outcomes of this trial were reported; improvements in some outcomes may have needed a longer-term to show up. Second, some outcomes were measured through the use of self-reported questionnaires, which could have introduced recall bias. Thirdly, some components of this intervention (e.g., transportation reimbursement and face to face heath education) were only provided for the duration of the intervention. Some benefits might not be expected to persist when those benefits end—for example, improvements in environmental QoL.

Despite these limitations, our findings have several implications for clinical practice and research. First, as demonstrated by the improvements in QoL in this population, the ILSM program could be recommended as an effective way to implement primary care service among women with prior GDM. Second, psychological outcomes (e.g., stress and social support) and personal physical health conditions should be considered when designing interventions oriented to change the physiological variables in this population. Third, studies with larger sample sizes using specialized measurements for psychological outcomes are needed. It is important to identify the intervention components that contribute to improvement in specific psychosocial outcomes. The mechanisms by which psychosocial outcomes were improved in the ILSM program are worthy of further analysis.

## 5. Conclusions

The ILSM program had favorable effects on the improvement of QoL in rural women with prior GDM, especially in their psychological and environmental domains. There was potential efficacy of the ILSM program on preventing the increasing trend of HbA1c, improving the physiological domain of QoL increasing subjective social support. The ILSM program provides a form of health promotion that could improve both the psychological and environmental health of QoL in rural women with prior GDM. The longer-term efficacy of the ILSM program is promising and will be reported elsewhere.

## Figures and Tables

**Table 1:** Baseline characteristics of women with gestational diabetes mellitus (GDM) by the randomization condition.

Characteristic	Total Sample	ILSM Group (*n* = 160)	Control Group (*n* = 160)	t/χ^2^	*p*
Age	31.92 (4.91)	32.16 (5.03)	31.69 (4.80)	−0.867	0.386
Ethnic				0.623	0.430
Han	175 (55.0%)	84 (52.8%)	91 (57.2%)		
Minority	143 (45.0%)	75 (47.2%)	68 (42.8%)		
Education level				0.001	0.973
Junior high school and below	78 (24.9%)	38 (24.8%)	40 (25.0%)		
Senior high school and above	235 (75.1%)	115 (75.2%)	120 (75.0%)		
Occupation				2.971	0.085
Part-time job or no job	123 (38.4%)	54 (33.8%)	69 (43.1%)		
Full-time job	197 (61.6%)	106 (66.2%)	91 (56.9%)		
Family monthly income per person				0.178	0.673
<$423	63 (19.7%)	30 (18.8%)	33 (2.6%)		
≥$423	257 (8.3%)	130 (81.3%)	127 (79.4%)		
Months after last delivery	17.55 (17.17)	17.38 (16.53)	17.73 (17.94)	0.148	0.882
≤12 months	141 (44.5%)	70 (44.9%)	71 (44.1%)	0.019	0.890
>12months	176 (55.5%)	86 (55.1%)	90 (55.9%)		
Parity	1.70 (.53)	1.67 (.54)	1.73 (.53)	0.912	0.363
1	76 (33.8%)	43 (36.8%)	33 (3.6%)	0.964	0.326
≥2	149 (66.2%)	74 (63.2%)	75 (69.4%)		
Perceived stress	22.78 (6.72)	22.75 (6.60)	22.82 (6.85)	0.100	0.920
Social support	51.59 (5.82)	51.28 (5.81)	51.90 (5.83)	0.812	0.418
Subjective support	27.14 (3.98)	27.20 (3.87)	27.08 (4.10)	−0.254	0.800
Objective support	15.86 (3.32)	15.62 (3.58)	16.11 (3.02)	1.298	0.195
Support utilization	8.22 (1.667)	8.28 (1.61)	8.17 (1.72)	−0.543	0.587
Physiological QoL	13.17 (1.69)	12.91 (1.63)	13.42 (1.71)	2.654	**0.008**
Psychological QoL	13.28 (1.93)	12.81(1.87)	13.75 (1.87)	4.446	**0.000**
Social relation QoL	14.91 (2.05)	14.69 (2.08)	15.14 (1.99)	1.931	0.054
Environment QoL	12.48 (1.80)	12.15 (1.59)	12.80 (1.94)	3.274	**0.001**
HbA1c	5.32 (0.43)	5.33 (0.39)	5.29 (0.45)	−0.786	0.433

Note: Continuous values are displayed as mean (SD) and categorical variables as frequency (%); significance was assumed when *p* < 0.05, as shown in bold.

**Table 2:** The effects of the intensive lifestyle modification (ILSM) intervention on perceived stress, social support, quality of life (QoL), and HbA1c.

Variables	ILSM Group			Control Group			*p*-Value		
	Baseline Mean (SD)	3 Months Mean (SD)	6 Months Mean (SD)	Baseline Mean (SD)	3 Months Mean (SD)	6 Months Mean (SD)	Time	Group	Group × Time
Perceived stress	22.75 (6.60)	24.22 (7.93)	24.18 (7.33)	22.82 (6.85)	24.53 (6.72)	24.60 (5.47)	0.925	0.194	0.739
Social support	51.28 (5.81)	49.25 (4.88)	5.64 (5.12)	51.90 (5.83)	49.18 (6.39)	52.00 (4.70)	0.714	0.945	0.509
Objective support	27.20 (3.87)	25.58 (3.75)	26.06 (3.90)	27.08 (4.10)	25.98 (3.80)	27.21 (3.19)	0.948	0.781	0.631
Subjective support	15.62 (3.58)	16.09 (3.29)	16.19 (3.31)	16.11 (3.02)	14.77 (4.16)	16.58 (2.96)	0.232	0.345	0.074
Support utilization	8.28 (1.61)	7.80 (1.52)	7.74 (1.64)	8.17 (1.72)	8.14 (1.71)	7.88 (1.68)	0.969	0.624	0.345
Physiological QoL	12.91 (1.63)	14.53 (2.01)	14.70 (2.02)	13.42 (1.71)	14.77 (1.73)	14.75 (1.83)	**0.032**	**0.010**	0.105
Psychological QoL	12.81(1.87)	14.20 (2.17)	14.24 (2.16)	13.75 (1.87)	14.46 (1.92)	14.26 (2.04)	0.342	**0.000**	**0.002**
Social relations QoL	14.69 (2.08)	14.82 (2.15)	15.08 (2.50)	15.14 (1.99)	15.41 (1.89)	15.27 (1.95)	0.868	**0.037**	0.439
Environment QoL	12.15 (1.59)	13.89 (2.02)	14.01 (2.13)	12.80 (1.94)	14.05 (1.98)	13.80 (2.05)	0.894	**0.000**	**0.001**
HbA1c	5.31 (0.46)	-	5.55 (0.34)	5.34 (0.39)	-	5.62 (0.33)	**0.000**	0.197	0.059

Note: significance was assumed when *p* < 0.05, as shown in bold.

## Data Availability

The data presented in this study are available on request from the corresponding author. The data are not publicly available due to privacy.

## References

[1] American Diabetes Association, 2 (2021). Classification and Diagnosis of Diabetes: Standards of Medical Care in Diabetes—2021. Diabetes Care.

[2] Zhu Y., Zhang C. (2016). Prevalence of Gestational Diabetes and Risk of Progression to Type 2 Diabetes: A Global Perspective. Curr. Diabetes Rep..

[3] Gao C., Sun X., Lu L., Liu F., Yuan J. (2019). Prevalence of gestational diabetes mellitus in mainland China: A systematic review and meta-analysis. J. Diabetes Investig..

[4] Crowther C.A., Hiller J.E., Moss J.R., McPhee A.J., Jeffries W.S., Robinson J.S. (2005). Effect of Treatment of Gestational Diabetes Mellitus on Pregnancy Outcomes. N. Engl. J. Med..

[5] Bellamy L., Casas J.-P., Hingorani A.D., Williams D. (2009). Type 2 diabetes mellitus after gestational diabetes: A systematic review and meta-analysis. Lancet.

[6] Tobias D.K., Fau H.F., Fau F.J., Fau C.J., Zhang C. (2011). Increased risk of hypertension after gestational diabetes mellitus: Findings from a large prospective cohort study. Diabetes Care.

[7] Nicklas J.M., Zera C.A., Seely E.W., Abdul-Rahim Z.S., Rudloff N.D., Levkoff S.E. (2011). Identifying postpartum intervention approaches to prevent type 2 diabetes in women with a history of gestational diabetes. BMC Pregnancy Childbirth.

[8] George L. (2005). Lack of preparedness: Experiences of first-time mothers. MCN Am. J. Matern. Child Nurs..

[9] Bennett W.L., Ennen C.S., Carrese J.A., Hill-Briggs F., Levine D.M., Nicholson W.K., Clark J.M. (2011). Barriers to and facilitators of postpartum follow-up care in women with recent gestational diabetes mellitus: A qualitative study. J. Womens Health.

[10] Van Ryswyk E., Middleton P., Shute E., Hague W., Crowther C. (2015). Women’s views and knowledge regarding healthcare seeking for gestational diabetes in the postpartum period: A systematic review of qualitative/survey studies. Diabetes Res. Clin. Pract..

[11] Feig D.S., Chen E., Naylor C.D. (1998). Self-perceived health status of women three to five years after the diagnosis of gestational diabetes: A survey of cases and matched controls. Am. J. Obstet. Gynecol..

[12] Post M.W. (2014). Definitions of quality of life: What has happened and how to move on. Top Spinal Cord Inj. Rehabil..

[13] Persson M., Winkvist A., Mogren I. (2015). Lifestyle and health status in a sample of Swedish women four years after pregnancy: A comparison of women with a history of normal pregnancy and women with a history of gestational diabetes mellitus. BMC Pregnancy Childbirth.

[14] Dalfrà M.G., Nicolucci A., Bisson T., Bonsembiante B., Lapolla A. (2012). Quality of life in pregnancy and post-partum: A study in diabetic patients. Qual. Life Res..

[15] Cohen S., Kamarck T., Mermelstein R. (1983). A global measure of perceived stress. J. Health Soc. Behav..

[16] Kaiser B., Razurel C., Jeannot E. (2013). Impact of health beliefs, social support and self-efficacy on physical activity and dietary habits during the post-partum period after gestational diabetes mellitus: Study protocol. BMC Pregnancy Childbirth.

[17] Dasgupta K., Da Costa D., Pillay S., De Civita M., Gougeon R., Leong A., Bacon S., Stotland S., Chetty V.T., Garfield N. (2013). Strategies to optimize participation in diabetes prevention programs following gestational diabetes: A focus group study. PLoS ONE.

[18] Thomas H. (2004). Women’s postnatal experience following a medically complicated pregnancy. Health Care Women Int..

[19] Evans M.K., Patrick L.J., Wellington C.M. (2010). Health Behaviours of Postpartum Women with a History of Gestational Diabetes. Can. J. Diabetes.

[20] Guo J., Chen J.L., Whittemore R., Whitaker E. (2016). Postpartum Lifestyle Interventions to Prevent Type 2 Diabetes Among Women with History of Gestational Diabetes: A Systematic Review of Randomized Clinical Trials. J. Womens Health.

[21] Pedersen A.L.W., Terkildsen Maindal H., Juul L. (2017). How to prevent type 2 diabetes in women with previous gestational diabetes? A systematic review of behavioural interventions. Prim. Care Diabetes.

[22] Bandura A. (2004). Health promotion by social cognitive means. Health Educ. Behav..

[23] Altazan A.D., Redman L.M., Burton J.H., Beyl R.A., Cain L.E., Sutton E.F., Martin C.K. (2019). Mood and quality of life changes in pregnancy and postpartum and the effect of a behavioral intervention targeting excess gestational weight gain in women with overweight and obesity: A parallel-arm randomized controlled pilot trial. BMC Pregnancy Childbirth.

[24] Guo J., Tang Y., Wiley J., Whittemore R., Chen J.L. (2018). Effectiveness of a diabetes prevention program for rural women with prior gestational diabetes mellitus: Study protocol of a multi-site randomized clinical trial. BMC Public Health.

[25] Faul F., Erdfelder E., Lang A.-G., Buchner A. (2007). G*Power 3: A flexible statistical power analysis program for the social, behavioral, and biomedical sciences. Behav. Res. Methods.

[26] Mensah G.P., ten Ham-Baloyi W., van Rooyen D., Jardien-Baboo S. (2020). Guidelines for the nursing management of gestational diabetes mellitus: An integrative literature review. Nurs. Open.

[27] Group W. (1994). Development of the WHOQOL: Rationale and Current Status. Int. J. Ment. Health.

[28] Yang T.-Z., Huang H.-T. (2003). An epidemiological study on stress among urban residents in social transition period. Chin. J. Epidemiol..

[29] Xiao S. (1994). Theoretical basis and research application of social Support Rating Scale. J. Clin. Psychiatry.

[30] Cheng Y., Liu C., Mao C., Qian J., Liu K., Ke G. (2008). Social support plays a role in depression in Parkinson’s disease: A cross-section study in a Chinese cohort. Parkinsonism Relat. Disord..

[31] Chinese Diabetes Society (2017). Guidelines for the prevention and control of type 2 diabetes in China. Chin. J. Diabetes.

[32] Ni Y., Liu S., Li J., Dong T., Tao L., Yuan L., Yang M. (2019). The Effects of Nurse-Led Multidisciplinary Team Management on Glycosylated Hemoglobin, Quality of Life, Hospitalization, and Help-Seeking Behavior of People with Diabetes Mellitus. J. Diabetes Res..

[33] Gilbert L., Gross J., Lanzi S., Quansah D.Y., Puder J., Horsch A. (2019). How diet, physical activity and psychosocial well-being interact in women with gestational diabetes mellitus: An integrative review. BMC Pregnancy Childbirth.

[34] Reinhardt J.A., van der Ploeg H.P., Grzegrzulka R., Timperley J.G. (2012). Implementing lifestyle change through phone-based motivational interviewing in rural-based women with previous gestational diabetes mellitus. Health Promot. J. Aust..

[35] Halkoaho A., Kavilo M., Pietilä A.-M., Huopio H., Sintonen H., Heinonen S. (2010). Does gestational diabetes affect women’s health-related quality of life after delivery?. Eur. J. Obstet. Gynecol. Reproduct. Biol..

[36] Philis-Tsimikas A., Fortmann A.L., Dharkar-Surber S., Euyoque J.A., Ruiz M., Schultz J., Gallo L.C. (2014). Dulce Mothers: An intervention to reduce diabetes and cardiovascular risk in Latinas after gestational diabetes. Transl. Behav. Med..

